# A foraminiferal δ^18^O record covering the last 2,200 years

**DOI:** 10.1038/sdata.2016.42

**Published:** 2016-06-21

**Authors:** Carla Taricco, Silvia Alessio, Sara Rubinetti, Gianna Vivaldo, Salvatore Mancuso

**Affiliations:** 1 Dipartimento di Fisica, Università di Torino, Torino 10125, Italy; 2 Osservatorio Astrofisico di Torino, INAF, Pino Torinese 10025, Italy; 3 IMT School for Advanced Studies, Lucca 55100, Italy

**Keywords:** Palaeoceanography, Palaeoclimate

## Abstract

Thanks to the precise core dating and the high sedimentation rate of the drilling site (Gallipoli Terrace, Ionian Sea) we were able to measure a foraminiferal δ^18^O series covering the last 2,200 years with a time resolution shorter than 4 years. In order to support the quality of this data-set we link the δ^18^O values measured in the foraminifera shells to temperature and salinity measurements available for the last thirty years covered by the core. Moreover, we describe in detail the dating procedures based on the presence of volcanic markers along the core and on the measurement of ^210^Pb and ^137^Cs activity in the most recent sediment layers. The high time resolution allows for detecting a δ^18^O decennial-scale oscillation, together with centennial and multicentennial components. Due to the dependence of foraminiferal δ^18^O on environmental conditions, these oscillations can provide information about temperature and salinity variations in past millennia. The strategic location of the drilling area makes this record a unique tool for climate and oceanographic studies of the Central Mediterranean.

## Background & Summary

Knowledge of natural climate variability is required to understand recently observed trends and to assess anthropogenic effects on climate. Instrumental series, however, cover only the last few centuries. Marine cores with very high sedimentation rates allow for investigating climate variations on scales of decades up to millennia.

For many years the Torino Cosmogeophysics group has studied sediment cores drilled from the Gallipoli Terrace in the Gulf of Taranto (Ionian Sea) and deposited in the last millennia. This site is close to the volcanic Campanian area, and this allowed us to date the cores with great accuracy by tephroanalysis. It also has a high sedimentation rate, allowing for a high time resolution (3.87 years).

We measured the oxygen isotope composition δ^18^O of planktonic foraminifera in one of the cores extracted from the Gallipoli Terrace. These measurements provided a high-resolution, 2,200-year-long record (see Data Citation 1) which allowed us to demonstrate that the millennial trend and the bicentennial oscillation detected in this series are temperature driven^1^.

In a recent paper^2^, using these isotopic data together with historical Po River discharge data and oceanographic measurements, we have shown that the Po River discharge undergoes robust decadal fluctuations that reach the Ionian Sea, ~1,000 km south of the Po River delta, by salinity anomalies propagation. These salinity variations have been registered in the foraminifera shells^3,4^ and allowed for the first time a reconstruction of North Italian hydrological variability on millennial-scale.

The value of this isotopic δ^18^O record, lying in its uncommonly high time-resolution and length, makes it worth of a thorough description. Therefore, here we provide details about the sediment core and its dating, as well as about the procedures of sample preparation and measurement. Moreover, in the last section we give several analysis and arguments validating the data-set.

## Methods

### Drilling area and characteristics of the core

**Figure 1:**
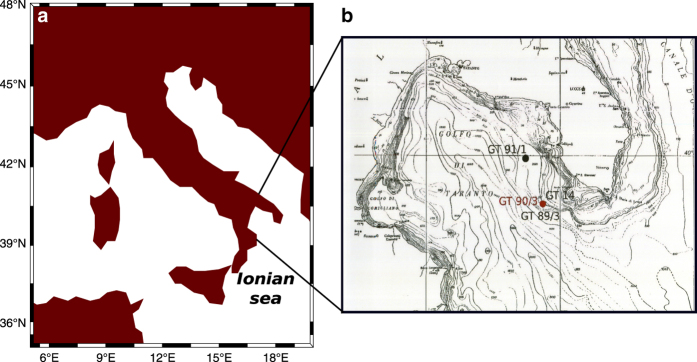
Location of the extraction site of core GT90/3. (**a**) Core GT90/3 was drilled in the Gulf of Taranto, Ionian Sea. The map was made with the Language for technical computing MATLAB, version R2015b (MathWorks Inc.) (**b**) Bathymetric map showing the Gallipoli Terrace, from which our cores were extracted. We measured δ^18^O in the GT90/3 core (red point).

The gravity core GT90/3, in which the δ^18^O series was measured, was drilled from the Gallipoli Terrace in the Gulf of Taranto (Ionian Sea) at 39°45′53′′N, 17°53′33′′E (see Fig. 1). It was extracted at a depth of 178 m and its length is 3.57 m. The map in Fig. 1 shows the extraction site of the GT 90/3 core and of the other ones we retrieved from the same Gallipoli Terrace (cores GT14 and GT89/3 (refs 5,6) were drilled very close to GT90/3 and are represented by the same point on this map). The cores are stored at the underground Laboratory of Monte dei Cappuccini (OATo-INAF) in Torino (Italy).

Thanks to its geographical location, the Gallipoli Terrace is a favourable site for climatic studies based on marine sediments, because of its closeness to the volcanically active Campanian area, a region that is unique in the world for its detailed historical documentation of volcanic eruptions (see the Technical Validation section). Tephra layers corresponding to historical eruptions were identified along the cores, thus allowing for accurate dating and determination of the sedimentation rate. The measurements performed in different cores from the same area showed that the sedimentation rate is uniform across the whole Gallipoli Terrace^5,6^. Our group measured several profiles in the cores, including ^210^Pb and ^137^Cs activity, as well as pyroxene density (see the Technical Validation section), CaCO_3_ content^5–8^ and thermoluminescence^9,10^, in addition to oxygen and carbon isotopic ratios^1,2,7,11,12^.

### Sample preparation and δ^18^O measurement

We sampled the core using a spacing of 2.5 mm. Each sample of sediment (5 g) was soaked in 5% calgon solution overnight, then treated in 10% H_2_O_2_ to remove any residual organic material. Subsequently it was washed with a distilled-water jet through a sieve with a 150 μm mesh. The fraction >150 μm was kept and oven-dried at 50 °C. The planktonic foraminifera *Globigerinoides ruber* were picked out of the samples under a microscope. For each sample, 20–30 specimens were selected from the fraction comprised between 150 and 300 μm.

The use of a relatively large number of specimens for each sample reduces the isotopic variability of individual organisms, giving a more representative δ^18^O value. The stable isotope measurements were performed using a VG-PRISM mass spectrometer fitted with an automated ISO-CARB preparation device. Analytical precision based on internal standards was better than 0.1‰. Calibration of the mass spectrometer to VPDB scale was done using NBS19 and NBS18 carbonate standards.

### δ^18^O profile

**Figure 2:**
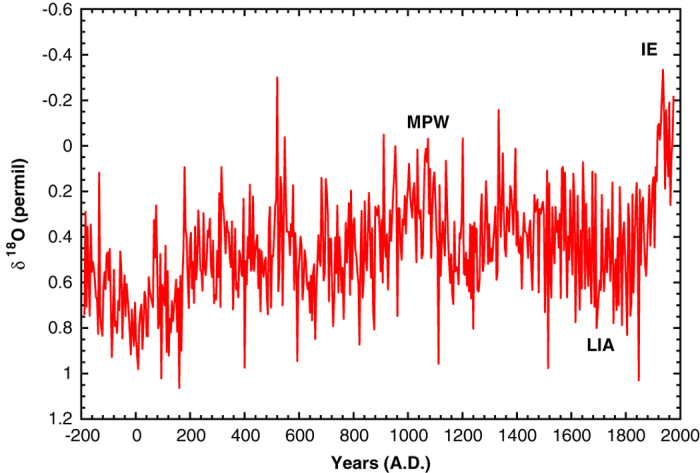
δ^18^O isotopic ratio profile measured in GT 90/3 core. Mean value is 0.46‰ and standard deviation is 0.23‰. The sampling interval is Δt=3.87 years and the data are expressed in VPDB standard. Since δ^18^O is inversely correlated with temperature, the δ^18^O series is plotted on a reversed y-axis.

The δ^18^O profile we obtained is shown in Fig. 2. It consists of a continuous record of 560 points from 200 BC to 1979 AD, with a sampling interval of Δt=3.87 y.

Several profile features clearly correspond to distinct climatic periods: the low δ^18^O values near 1000 AD are associated with the Medieval Warm Period (MWP) and to the Industrial Era (IE); relatively high values correspond to the Little Ice Age (LIA, 1600–1800 AD). We also find a δ^18^O maximum at about 0 AD, suggesting low temperature at that time (this controversial period is deeply discussed in Sec. 5 of Taricco *et al.* ^1^).

## Data Records

The δ^18^O record (Fig. 2), covering the last 2 millennia, is available on Pangaea (see Data Citation 1) in machine-readable ASCII format. The file has four columns, the first one with the depth of the sample (in cm), the second and the third ones with the date of the sample (in years AD and BP respectively) and the fourth with the δ^18^O value (permil).

## Technical Validation

### Uncertainties of δ^18^O measurements

Regarding the uncertainties of δ^18^O measurements, we did not replicate the isotopic analysis at a given depth in the core to estimate the uncertainty, because of the small number of *Globigerinoides ruber* in the sediment. In order to obtain a value of δ^18^O, which is representative of the sample, we need to collect about 20–30 specimens, which is about the total number of suitable foraminifera contained in each 2.5 mm-thick slice. For this reason, we prefer to choose high resolution and to extract later the signal from the noise, using statistical methods applied to the whole series. This is done by a Monte Carlo procedure that allows for assigning a confidence level to each variability mode detected in the series, as described in the next subsection.

### Extraction of signal from noise

The δ^18^O series was analysed by different spectral methods^1^. Using Singular Spectrum Analysis^13–16^(SSA) we demonstrated that the statistically significant part of the δ^18^O time series is given by the sum of components with periods of 770, 300, 180, 125 and 11.4 years, respectively (periods deduced by applying the Maximum Entropy Method (MEM)). The signal accounts for roughly 42% of the total variance in the series.

Monte Carlo-SSA^15^ allows us to verify that the statistically significant part (98% c.l.) of the δ^18^O time series is given by the sum of these five components, with a residue of red noise. We obtained this result after rejecting a whole range of null hypotheses, including different combinations of components.

Recently we focused on the decennial range of periods^2^ and we showed that the robust decadal component is related to the Po River discharge variations.

### High-frequency (decadal) variability in the δ^18^O series

The highest-frequency variation detected in the δ^18^O series is a decadal oscillation. This component is central in Taricco *et al.* ^2^, where it allowed for reconstructing the Po river discharge over two millennia. For this reason, it deserves a more in-depth discussion.

**Figure 3:**
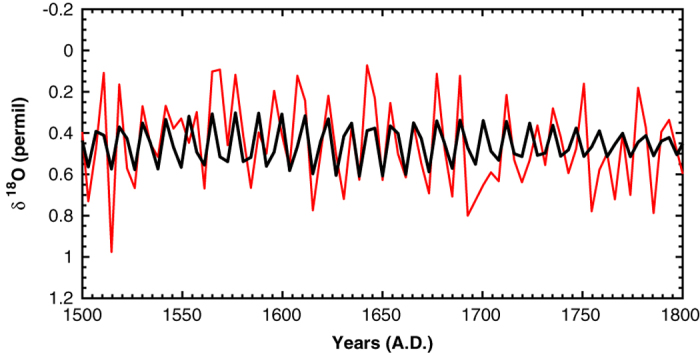
Comparison between the decadal component of the δ^18^O record (black curve) and the raw data (red curve).

Despite its short period with respect to the time resolution of the series, this oscillation is visible even looking at the raw data. This can be appreciated comparing (Fig. 3) the decadal variation reconstructed by SSA (black curve) and the raw data (red curve) over a part of the series.

The actual presence of this oscillation is supported by the following arguments. First, we detected it at high confidence level (99%); second, its period is not too close to the Nyquist period (7.7 years).

Moreover, it must be underlined that the experimental procedure rules out the issue of frequency aliasing. Indeed, the discretization of our series is not the result of a punctual sampling of a continuous signal, but derives from the measurement of consecutive sediment slices performed after mixing the material contained in each of them. This mixing removes any possible frequencies higher than the Nyquist frequency, thus acting as a low-pass filter, which avoids the frequency aliasing.

### Theoretical validation of the modern experimental δ^18^O values

As is common knowledge, planktonic foraminifera are strongly influenced by the environmental conditions of the near-surface sea in which they live, particularly due to their sensitivity to environmental temperature and salinity.

The theoretical (expected) δ^18^O_c_ in the foraminiferal calcite was determined at different depths using the relationship with temperature (T) and δ^18^O_w_ of the water, given by Shackleton^3^,

(1)δ18Oc=3.86+δ18Ow−0.23T

and the relationship derived by Pierre^4^ for Mediterranean surface waters (S is the salinity of the considered layer),

(2)δ18Ow=0.25S−8.2

**Figure 4:**
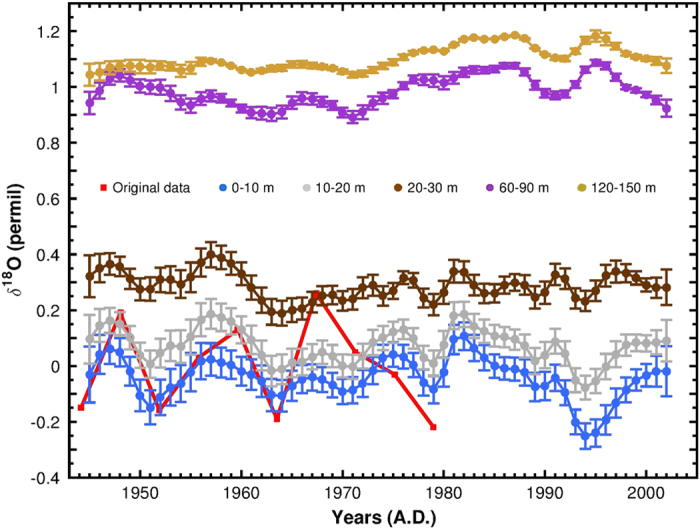
Comparison between measured Globigerinoides ruber of the core GT 90/3 δ^18^O (red points) and theoretical values of calcite δ^18^O (from Taricco *et al.*, 2015^2^). The expected δ^18^O series and the corresponding 1-standard-deviation uncertainties were obtained using the Shackleton equation with salinity and temperature yearly-resolved data at different depths (from ref. 18), corresponding to the same Gallipoli site (plotted values are anomalies with respect to the average value of the 0–20 m layer).

By combining these equations it was possible^2^ to calculate δ^18^O at different depths using observed salinity and temperature^17,18^ in the vicinity of the core location. Figure 4 compares the measured δ^18^O values with the calculated ones over the common period 1945–1979 covered by both the series of foraminiferal δ^18^O and the sea temperature/salinity ones.

Here we recall the results obtained by this comparison and discussed in Taricco *et al.*^2^

The five theoretical δ^18^O series of Fig. 4 show that: a) decadal variations are present in the upper layers; b) in spite of the different temporal resolution, these variations are in phase with the foraminiferal δ^18^O (red points), and the experimental amplitudes of the variations (0.2–0.3 permil) are in good agreement with the theoretical estimates for the layer in which these organisms dwell, i.e., 0 to 20 m depth.

### Core dating

The δ^18^O series here described is particularly valuable due to the accurate dating of the core. It is thus worth recalling the tephroanalysis and radiometric measurements that led us to determine the sedimentation rate of the Gallipoli Terrace.

#### Tephroanalysis

**Figure 5:**
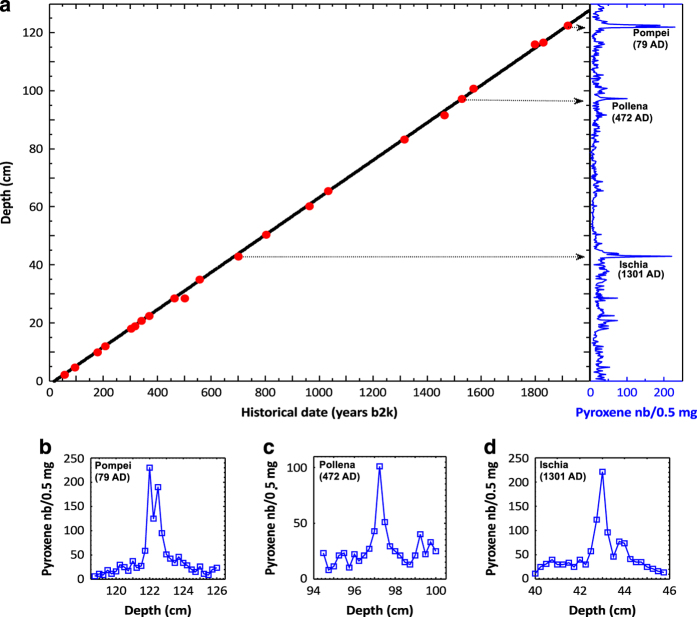
Time-depth calibration of the core. (**a**) The depth at which a volcanic peak is found in the sediment is plotted versus the historical date of the corresponding eruption, expressed in years counted backwards from 2000 AD. Each red dot in the figure represents a volcanic eruption, corresponding to a peak in the clinopyroxene time series: 22 historical volcanic eruptions occurred in the Campanian area during the last two millennia and have been identified along the core. The straight line resulting from a linear regression fit to the experimental data is also shown (black line). In the right part of panel a, the pyroxene number record is plotted. The arrows indicate the correspondence between the most prominent peaks of the record and the associated points on the calibration line. (**b**–**d**) Peaks of the pyroxene number record corresponding, respectively, to the eruptions of Pompei, Pollena and Ischia. These peaks are those indicated by the arrows in panel (**a**).

The high accuracy of the dating of Ionian Sea cores was made possible by the closeness of the drilling site to the Campanian area, a region which is unique in the world for its detailed historical documentation of volcanic eruptions over the last two millennia^19^. The markers of these eruptions were identified along the cores as peaks of the number density of clinopyroxenes crystals. We found 22 sharp pyroxene peaks, corresponding to historical eruptions of the Campanian area, starting with the 79 AD eruption of Vesuvius that buried Pompei and ending with the last one in 1944 (see Table 1). Figure 5, panel a, shows the time-depth relation over the last two millennia.

Each point represents a pyroxene peak found at a given depth and corresponding to a historical eruption. The linear regression (correlation coefficient r=0.99) gives a sedimentation rate of (0.0645±0.0002)cm y^−1^. This relatively high value allows for high time-resolution studies: the sampling interval of 2.5 mm corresponds to 3.87 years. The highly linear time-depth relation demonstrates that the sedimentation rate has remained constant, to a really good approximation, over the last two millennia. Moreover, the measurements performed in different cores retrieved from the same area showed that this rate is also uniform across the whole Gallipoli Terrace^5–7^. Taricco *et al.*^20^ confirmed this dating by applying advanced statistical procedures^21,22^. The tephroanalysis dating confirmed, improved and extended to the deeper part of the core the dating obtained in the upper 20 cm by the ^210^Pb method^23,24^.

#### Radiometric core dating

**Figure 6:**
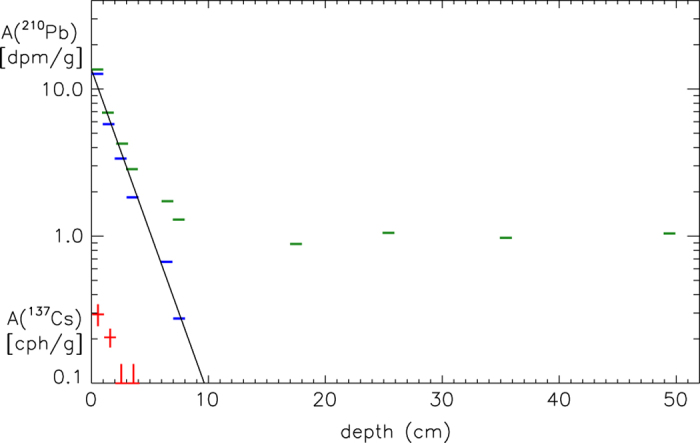
^210^Pb activity as a function of sediment depth (from Bonino *et al.*^23^). The sediment samples for the activity estimation were taken at 10 different depths, with a sampling thickness of 1 cm. Green and blue hyphens are the total and excess activity, respectively. The black line is the least-square fit of the excess activity. ^137^Cs activity is reported in the left-hand bottom corner of the figure (in red).

The ^210^Pb activity evaluation^23^ was carried out at the low-level counting Laboratory of ‘Monte dei Cappuccini’ (OATo-INAF) in Torino (70 m.w.e.). The activity was determined through the detection of the β^−^ activity of the ^210^Bi (T_1/2_=5.013 d) in equilibrium with ^210^Pb, using a set of Geiger-Muller counters operating inside a shielded well. The experimental measurements are shown in Fig. 6.

The decreasing solid line, which represents the least-square exponential fit to the excess activity, allowed to deduce the sedimentation rate S=0.064 cm y^−1^, with an estimated error of 5% and a correlation coefficient r=0.99 (ref. 23). Moreover, in order to validate the results obtained by the ^210^Pb method and to test the presence of the core top, Bonino *et al.*^23^ measured ^137^Cs activity (T_1/2_=30 yr), which is primarily due to nuclear bomb testing and related to the radioisotope concentration maximum in the atmosphere that occurred during the 1960s. The γ-activity measurement of ^137^Cs (see Fig. 6) was performed using a HPGe detector with relative efficiency of 25%, located at the underground Laboratory of ‘Monte dei Cappuccini’ in Torino. The activity maximum was revealed in the first sample, as expected on the basis of ^210^Pb dating. This result ensured that the core top is present and has not been disturbed during drilling operations or by biological activity, at least within the sampling thickness.

### Limited bioturbation of the sediment and presence of the core top

The very sharp pyroxene peaks (see, for example, Fig. 5—panels b,c and d showing the peaks corresponding to the Pompei, Pollena and Ischia eruptions) indicate that bioturbation by bottom-dwelling organisms is quite limited. Therefore, this climatic record is not significantly affected by sediment mixing. Moreover, the presence of the core top is demonstrated by the ^137^Cs activity, as described in the previous paragraph.

### Validity of the dating over the whole Gallipoli Terrace

The profiles of CaCO_3_ content measured in different cores retrieved from the same area showed that the sedimentation rate is uniform across the whole Gallipoli Terrace^5–8^. The CaCO_3_ content of each layer was determined by titration with EDTA, using the procedure explained by Barnes^25^.

The profiles of the CaCO_3_ content of different cores display a remarkable layer-to-layer correspondence, within a fraction of a percent. This unusual feature guarantees an essential requisite for the reproducibility and generality of our results, i.e., the uniform stratigraphy of the Gallipoli Terrace.

## Additional Information

**How to cite this article:** Taricco, C. *et al.* A foraminiferal δ^18^O record covering the last 2,200 years. *Sci. Data* 3:160042 doi: 10.1038/sdata.2016.42 (2016).

## Supplementary Material



## Figures and Tables

**Table 1:** Historical date and corresponding pyroxene-peak depth of the 22 eruptions identified along the core.

**historical date (yr AD)**	**pyroxene peak depth (cm)**	**eruption name**
79	122.5	Pompei
172	116.75	
203	116	
430	100.75	
472	97.25	Pollena
536	91.75	
685	83.25	
968	65.5	
1037	60.25	
1198	50.5	
1301	43	Ischia
1444	35	
1500	28.5	
1538	28.5	
1631	22.5	
1660	20.75	
1682	19	
1698	18	
1794	12	
1822	10	
1906	4.75	
1944	2.25	
